# Diabetes Stimulates Osteoclastogenesis by Acidosis-Induced Activation of Transient Receptor Potential Cation Channels

**DOI:** 10.1038/srep30639

**Published:** 2016-07-29

**Authors:** Carlotta Reni, Giuseppe Mangialardi, Marco Meloni, Paolo Madeddu

**Affiliations:** 1 Division of Experimental Cardiovascular Medicine, Bristol Heart Institute, University of Bristol, UK; 2 Vascular Pathology and Regeneration, Bristol Heart Institute, University of Bristol, UK; 3 University/British Heart Foundation Centre for Cardiovascular Science, The Queen’s Medical Research Institute, University of Edinburgh, UK

## Abstract

Patients with type 1 diabetes have lower bone mineral density and higher risk of fractures. The role of osteoblasts in diabetes-related osteoporosis is well acknowledged whereas the role of osteoclasts (OCLs) is still unclear. We hypothesize that OCLs participate in pathological bone remodeling. We conducted studies in animals (streptozotocin-induced type 1 diabetic mice) and cellular models to investigate canonical and non-canonical mechanisms underlying excessive OCL activation. Diabetic mice show an increased number of active OCLs. *In vitro* studies demonstrate the involvement of acidosis in OCL activation and the implication of transient receptor potential cation channel subfamily V member 1 (TRPV1). *In vivo* studies confirm the establishment of local acidosis in the diabetic bone marrow (BM) as well as the ineffectiveness of insulin in correcting the pH variation and osteoclast activation. Conversely, treatment with TRPV1 receptor antagonists re-establishes a physiological OCL availability. These data suggest that diabetes causes local acidosis in the BM that in turn increases osteoclast activation through the modulation of TRPV1. The use of clinically available TRPV1 antagonists may provide a new means to combat bone problems associated with diabetes.

Diabetes Mellitus (DM) represents one of the major threats to human health. Among acknowledged complications of DM, altered bone homeostasis is under-appreciated^1^,^2^. Augmented bone fragility is often associated with normal or even increased bone mineral density in patients with type 2 DM (DM2)^3^,^4^. In contrast, type 1 DM (DM1) is frequently accompanied by low bone density and osteoporosis, responsible for a 7 to 12-fold increased risk of fractures compared to subjects without DM. In a recent study, the annual cost of treating osteoporotic fractures has been estimated $19 billion, an amount that is expected to double over by 2025^5^. Noteworthy, the presence of DM in a patient with a hip fracture is a risk factor for increased mortality. However, current treatment regimens employed to prevent osteoporosis and accelerate fracture healing have not been specifically tested in patients with DM. Therefore, osteoporosis remains an unmet clinical need in diabetic patients.

To date, attention has been focused on osteoblasts^6^,^7^, whereas osteoclasts received less attention. Seminal studies have proposed an increased osteoclast activity in DM1^8^,^9^,^10^ but a clear mechanism behind this phenomena has yet to be elucidated. Moreover hyperglycemia, a noticeable factor that directly affects osteoblast function and bone formation^11^, cannot be the only link between osteoporosis and DM. In fact, recent studies demonstrate an inhibitory effect of high glucose on osteoclast activation^12^,^13^. A meta-analysis study showed that glycated haemoglobin, a measure of glycemic control, is not linked to osteoporosis^14^. Additionally, if the relationship between osteoporosis and DM is only related to hyperglycemia, the incidence of low mineral density should be similar in patients with DM1 and DM2.

This study was designed to evaluate the contribution of osteoclast activation in bone remodeling using the strepotozocin (STZ)-induced DM1 mouse model. *In vitro* and *in vivo* experiments were conducted to explore canonical and non-canonical pathways of osteooclastic activation^15^. In particular, we focused on Receptor Activator of Nuclear Factor Kappa-B Ligand (RANKL), osteoprotegerin (OPG), Tumor Necrosis Factor (TNF) and Transient Receptor Potential (TRP) cation channels related pathways. We discovered that local acidosis represents a predominant trigger of TRP cation channels-induced osteoclast activation in DM1. Blockade of this mechanism prevents osteoclast activation *in vivo*, thus opening new avenues for treatment of a common complication of DM.

## Results

### DM1 induces osteoclast activation

**Figure 1:**
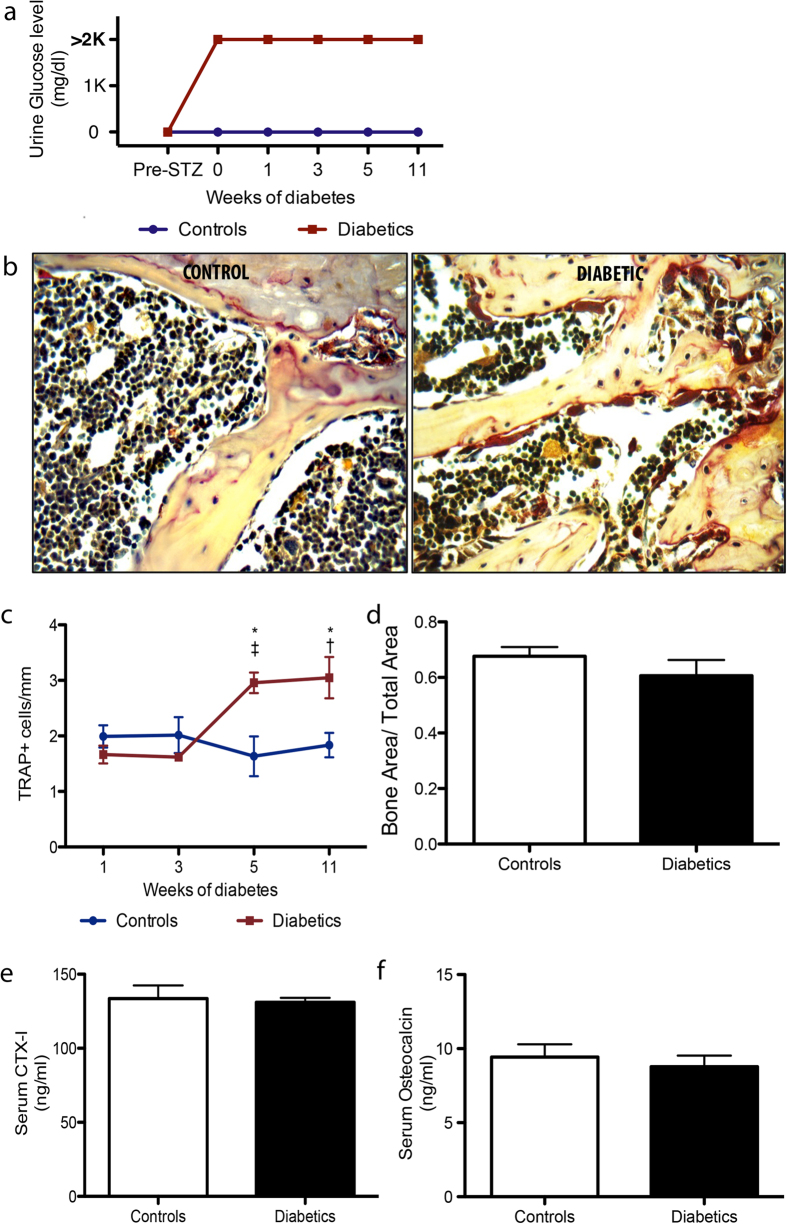
Diabetes induces osteoclast activation. (**a**) Graph shows urine glucose levels in control and diabetic animals. (**b**) Representative microphotographs of TRAP^pos^ osteoclasts at 5 weeks of DM. Scale bar: 20 μm. (**c**) Graph shows the kinetics of osteoclast activation up to 11 weeks of DM. Data (mean ± SEM) express the number of TRAP^pos^ osteoclasts/mm of endosteal bone. (**d**) Graph shows the ratio between bone and total tissue area at 5 weeks of DM. Charts representing the levels of bone resorption product CTX-I (**e**) and osteocalcin (**f**) in PB plasma at 5 weeks of DM. *p < 0.05 Diabetics vs Controls; ^†^p < 0.05 vs 1 and 3 weeks of DM. Studies were performed on 4 mice per group.

An experimental model of STZ-induced DM1 in mice was employed to verify the effect of DM1 on osteoclast activation. The diabetic status was confirmed by the presence of persistent glycosuria (Fig. 1a). We first compared the abundance of active osteoclasts in the bone marrow (BM) of STZ-induced diabetic mice and age-matched non-diabetic controls. Osteoclasts activation was assessed by histochemical analyses for tartrate resistant acid phosphatase (TRAP). As shown in Fig. 1b,c, the number of TRAP^pos^ osteoclasts lining the endosteal surface in metaphyseal and epiphyseal region was remarkably increased at 5 and 11 weeks from DM induction.

Interestingly, no differences were noted in bone volume (Fig. 1d) or in systemic markers of bone turnover (CTX-I and osteocalcin) (Fig. 1e,f) of STZ-induced diabetic mice and age-matched non-diabetic controls. These results indicate that early stages of diabetes are not yet characterised by changes in bone phenotype and systemic markers associated with bone remodelling.

### DM-induced osteoclast activation is RANKL/OPG independent

The contribution of canonical RANKL/OPG pathway was assessed at different time-points (5 and 11 weeks) by measuring the protein levels (ELISA assay) of those opposing factors in the BM supernatants and peripheral blood (PB) of diabetic and control mice. As shown in Fig. S1a, DM1 significantly reduced RANKL levels in both BM and PB, although with a different timing in the two compartments. Moreover, OPG levels were increased in PB of diabetic mice at 5 weeks from DM1 induction as compared with controls, but did not differ in BM of the two studied groups (Fig. S1b). Consequently, DM1 induced a reduction in the RANKL/OPG ratio (Fig. S1c).

### DM1 does not affect the expression of beta2-integrin, a transcriptional suppressor of osteoclastogenesis

In order to verify if suppression of CD11b, which acts as a negative regulator of the earliest stages of osteoclast differentiation, may account for the increased number of osteoclasts in diabetic BM, we next analysed the expression of CD11b/CD18 β2 integrin in total and endosteal BM cells using flow cytometry. Results indicate that DM1 does not affect the number of BM CD11b cells (Fig. S2a) or the percentage of dim and bright populations among the CD11b positive cells (Fig. S2b).

### Role of hypoxia and metabolic acidosis in DM1-induced osteoclast activation

**Figure 2:**
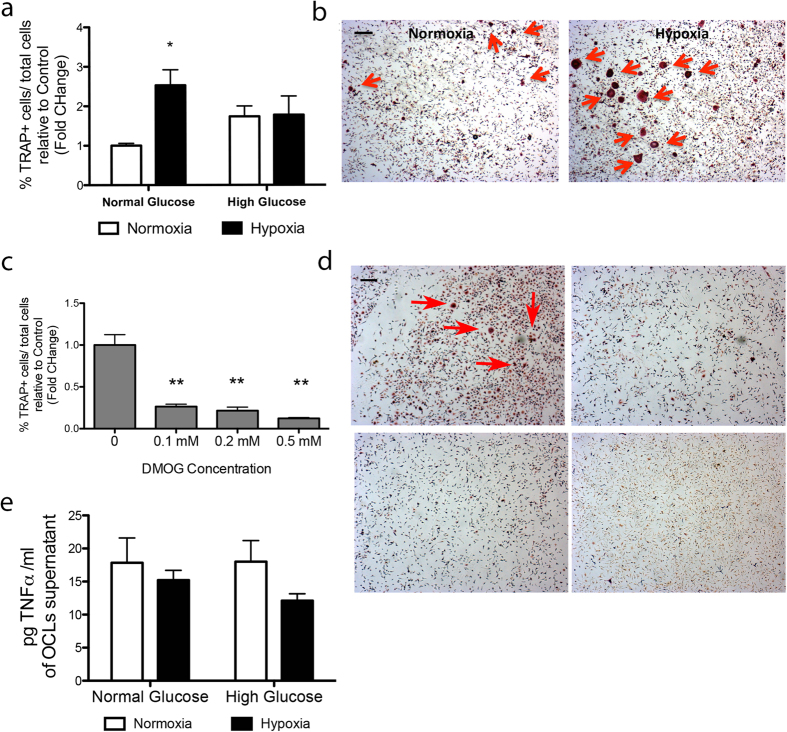
Effects of HG and hypoxia on osteoclast differentiation and activation *in vitro*. Bar graphs (**a**) and representative microphotographs (**b**) show the abundance of TRAP^pos^ osteoclasts derived from BM-MNCs cultured under normal/high glucose and normoxic/hypoxic conditions. Bar graphs (**c**) and representative microphotographs (**d**) show the abundance of TRAP^pos^ osteoclasts derived from BM-MNCs exposed to the hypoxia mimic DMOG. Data are expressed as mean ± SEM. *p < 0.05 vs normoxia and **p < 0.01 vs 0 mM DMOG. n = 3 samples/group. Scale bar: 200 μm. Bar graphs show the level of TNFα in supernatants of BM-MNCs cultured in normoxia/hypoxia and normal/high glucose (**e**). n = 4 samples/group.

**Figure 3:**
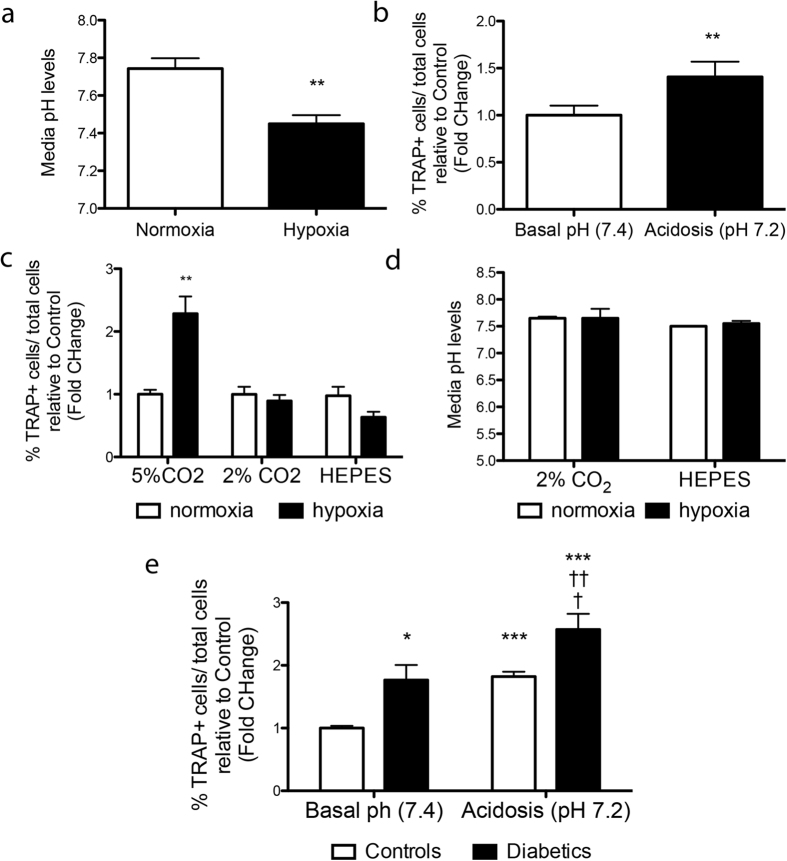
Role of acidosis in hypoxia-induced osteoclast activation: pH levels in media collected from BM-MNCs cultured under hypoxia or normoxia (**a**) or in the presence of buffering systems to prevent acidosis (**c**). Bar graph shows the abundance of TRAP^pos^ osteoclasts derived from BM-MNCs cultured under basal or acidosis conditions (**b**) or following addition of 2% CO_2_ or HEPES to buffer hypoxia-induced acidosis (**d**). Bar graph shows the abundance of TRAP^pos^ osteoclasts derived from BM-MNCs of healthy or diabetic animals (**e**). Data are expressed as mean ± SEM and **p < 0.01 vs. normoxia and basal pH; *p < 0.05 vs. Basal pH controls, ***p < 0.001 vs Basal pH controls, ^†^p < 0.05 vs pH 7.2 controls, ^††^p < 0.05 vs Basal pH diabetes. n = 6 samples/group for A, 18 samples/group for (**b**), and 4 samples/group for (**c**–**e**).

We next investigated the involvement of hypoxia in osteoclast differentiation and activation. Results illustrated in Fig. 2a,b confirm hypoxia is a potent inducer of *in vitro* osteoclastogenesis, although non-additive to high glucose. We also examined the effects of the hypoxia mimetic dimethyloxallyl glycine (DMOG), which enhances HIF-1 levels by inhibiting prolyl-4-hydroxylase domain enzymes. Results clearly demonstrate that DMOG reduces the differentiation of BM-mononuclear cells (MNCs) into osteoclasts (Fig. 2c,d), thus suggesting that osteoclast differentiation and activation is not directly triggered by an HIF-1 dependent mechanism. Furthermore, the protein levels of TNFα (Fig. 2e) and IL-1α and β (data not shown) did not differ in supernatants of osteoclasts cultured under normoxia/hypoxia and normal/high glucose conditions, thus suggesting these chemokines are not directly involved in osteoclasts activation triggered by low oxygen or high glucose. However, the exposure of BM-MNCs to hypoxia causes a reduction in the pH of culture media (Fig. 3a). Therefore, the implication of acidosis in osteoclast differentiation was further assessed. Under normoxic conditions, the differentiation of BM-MNCs into TRAP^pos^ osteoclasts increases by lowering the pH of the culture medium (Fig. 3b). In addition, pH stabilization by decreasing the CO_2_ tension from 5% to 2% CO_2_ or adding HEPES to the culture system inhibits hypoxia-induced osteoclast activation (Fig. 3c,d), thus excluding the direct involvement of hypoxia and underlying the role of acidosis in osteoclast differentiation. Noteworthy, the increased osteoclastogenic propensity of BM-MNCs from diabetic mice was further enhanced under induced *in vitro* acidosis (Fig. 3e).

### DM1 induces local acidosis in the BM, which is not reverted by insulin replacement

**Figure 4:**
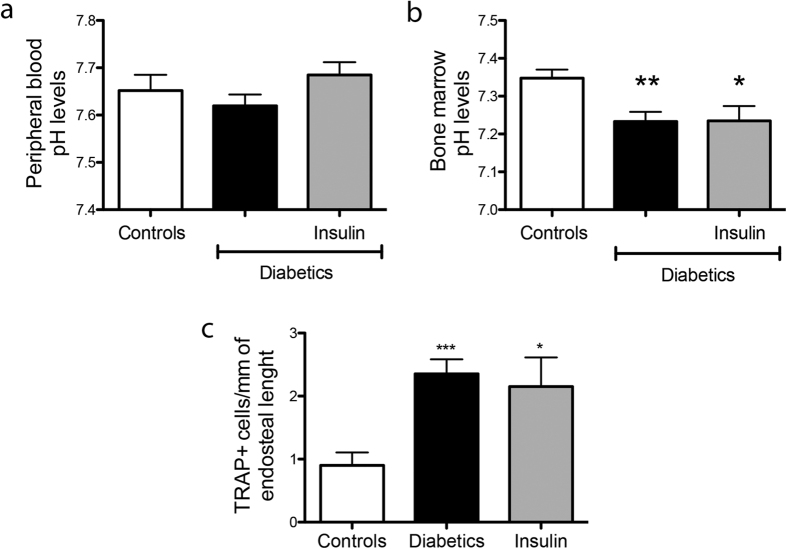
Insulin replacement does not revert acidosis and osteoclastogenesis. Graph shows pH of PB (**a**) and BM supernatants (**b**) from mice with STZ-induced DM given insulin replacement (grey bars) or vehicle (black bars) as compared with non-diabetic control mice (white bars). Graph shows the number of TRAP^pos^ osteoclasts/mm of endosteal bone (**c**). Data are expressed as mean ± SEM. *p < 0.05, **p < 0.01 and ***p < 0.001 vs. non-diabetic; n = 6 samples/group.

Interestingly, DM1 does not alter the pH of PB (Fig. 4a), but reduces it in BM supernatants (Fig. 4b), thus suggesting a specific dysregulation of mechanisms controlling physiologic pH in the marrow compartment. Chronic infusion of insulin achieved metabolic control as assessed by measurement of glycosuria (data not shown). However, insulin replacement did not correct the pH levels in the BM supernatant of diabetic mice (Fig. 4b). In line with these results, the abundance of active osteoclasts in diabetic mice receiving insulin replacement was not reverted to the level of healthy controls (Fig. 4c).

### Hypoxia-induced acidosis triggers osteoclast differentiation and activation through modulation of tripv1 ion channel

**Figure 5:**
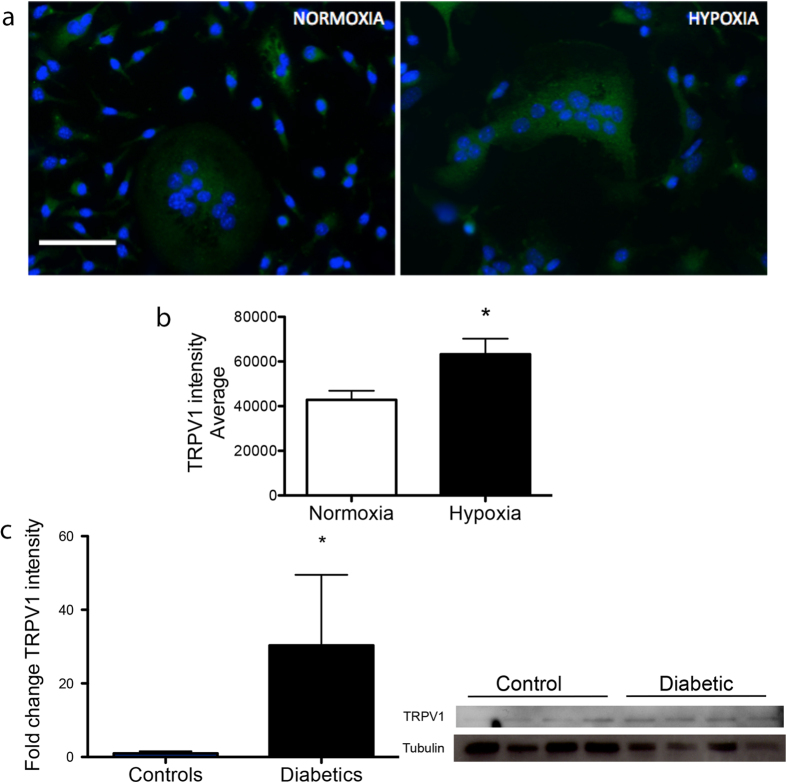
Expression of TRPV1 ion channel in diabetes. Representative microphotographs displaying TRPV1 staining (green) in BM-derived osteoclasts in normoxic and hypoxic conditions. Nuclei are stained with dapi (blue). Scale bar: 50 μm. (**a**) Graph illustrates the increase of TRPV1 intensity in cells cultured in hypoxia as compared to normoxia. Data are expressed as the average of TRPV1 intensity per single cells, corrected for the background (**b**). Representative western blot and graph showing TRPV1 expression in BM-MNCs isolated from diabetic and control mice. Tubulin was used for normalization (**c**). Data are expressed as mean ± SEM. *p < 0.05 vs normoxia and non-diabetic controls; n = 4 samples/group.

Acidosis-induced formation and activation of osteoclasts is known to be influenced by the activation of TRP cation channels^16^,^17^. We next investigate the involvement of TRPV1 cation channel in hypoxia/acidosis both *in vitro* and *in vivo*. Immunocytochemical analysis shows that TRPV1 expression is significantly upregulated by hypoxia in osteoclasts from non-diabetic mice (Fig. 5a,b). In addition, TRPV1 protein levels (assessed by western blot) are remarkably higher in diabetic BM-MNCs as compared with non-diabetic BM-MNCs (Fig. 5c)

**Figure 6:**
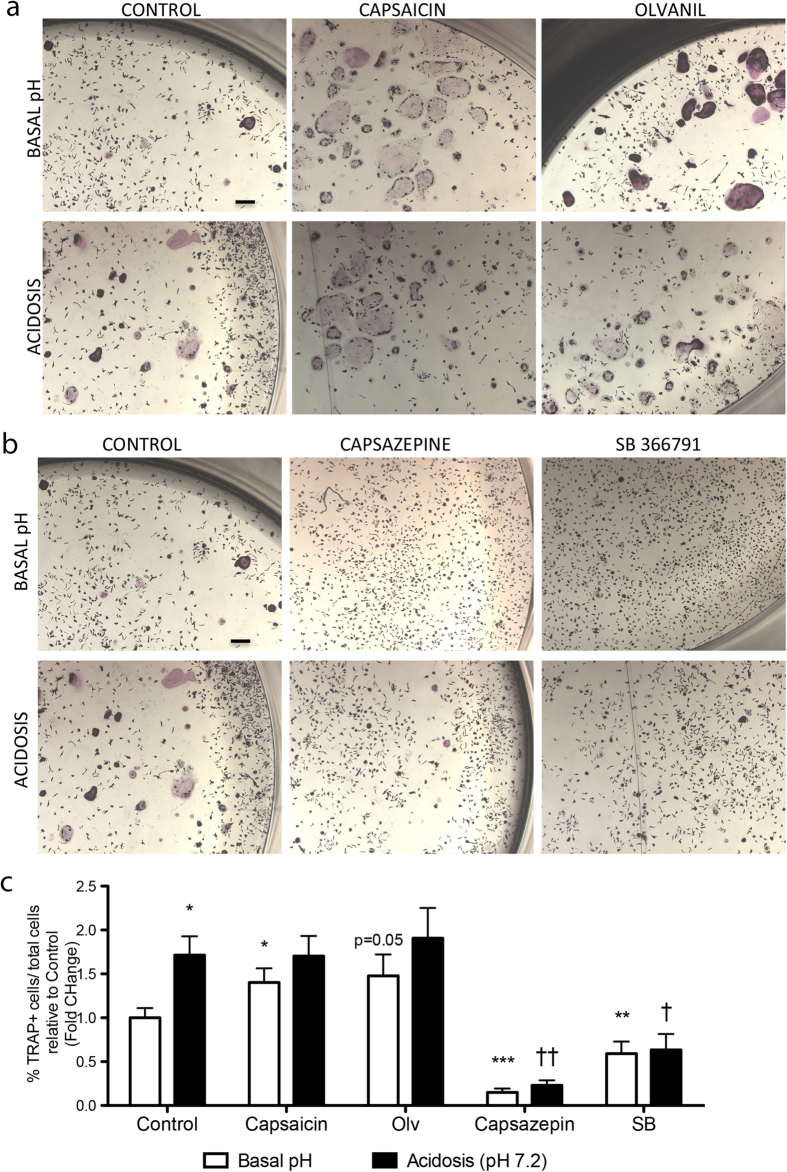
Effects of TRPV1 agonists and antagonists on osteoclast differentiation. Representative microphotographs (**a**,**b**) and bar graphs (**c**) show the abundance of TRAP^pos^ osteoclasts derived from BM-MNCs cultured with TRPV1 agonists (**a**) capsaicin (10 μM) or olvanil (10 μM) and antagonists (**b**) capsazepine (10 μM) or SB-366791 (10 μM) under normal (7.4) or low (7.2) pH. Data are expressed as mean ± SEM. *p < 0.05 and **p < 0.01 and ***p < 0.001 vs control at basal pH, ^†^p < 0.05 and ^††^p < 0.01 vs control acidosis. n = 7 samples/group. Scale bar: 200 μm.

**Figure 7:**
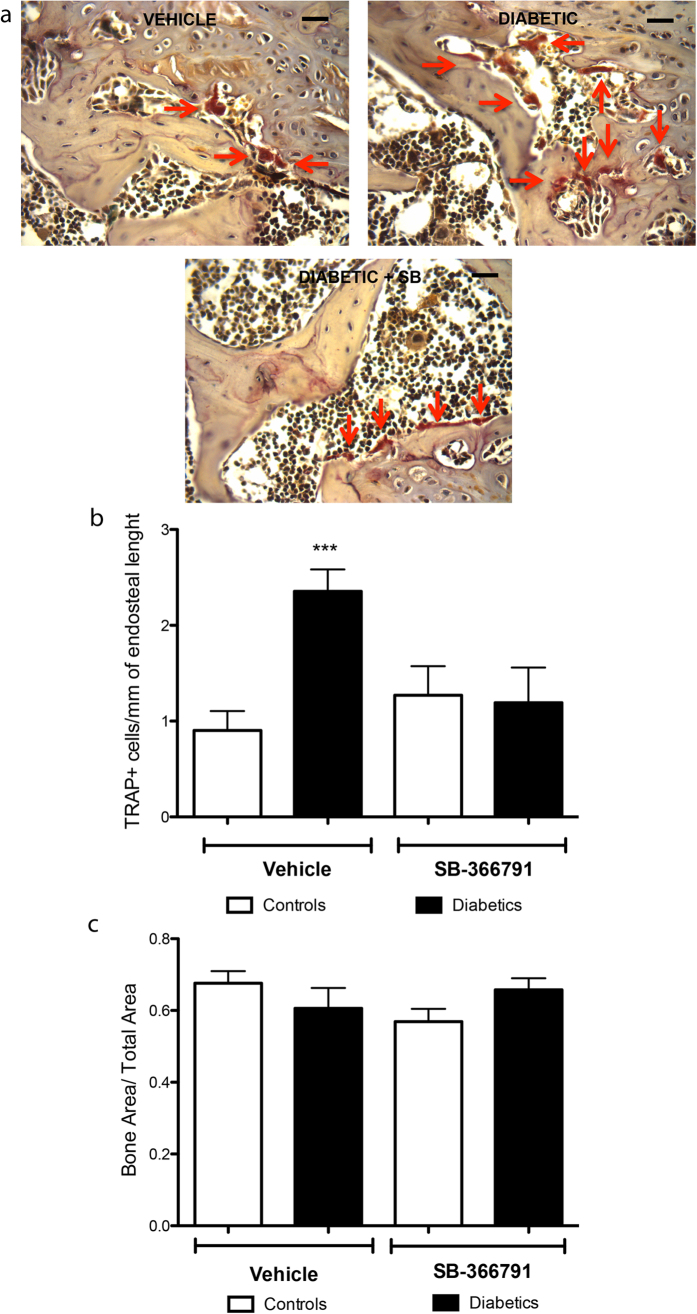
TRPV1 antagonist SB-366791 prevents osteoclast formation in diabetic animals. Representative microphotographs (**a**) and bar graph (**d**) show the abundance of TRAP^pos^ osteoclasts/mm of endosteal bone of controls and diabetic animals (5 weeks) treated with vehicle or TRPV1 antagonist SB-366791. Scale bar: 20 μm. Data are expressed as mean ± SEM. ***p < 0.001 vs vehicle controls. n = 6 animals/group.

Importantly, *in vitro* activation of TRPV1 by using its agonist capsaicin or olvanil induces differentiation of BM-MNCs into osteoclasts under normal pH levels, whereas no additional effects were observed under acidosis (Fig. 6a,c). Conversely, differentiation of osteoclasts was significantly decreased after inhibition of TRPV1 by using its antagonists, capsazepine or SB-366791, under normal or acidic pH (Fig. 6b,c). To further confirm the involvement of TRPV1 in DM!-induced osteoclast activation, healthy and diabetic animals were treated with TRPV1 antagonist SB-366791. Data shows that the antagonist reduces the number of TRAP^pos^ cells in diabetic animals to normal levels (Fig. 7), thus indicating the participation of TRPV1 in DM1-induced osteoclast activation.

## Discussion

**Figure 8:**
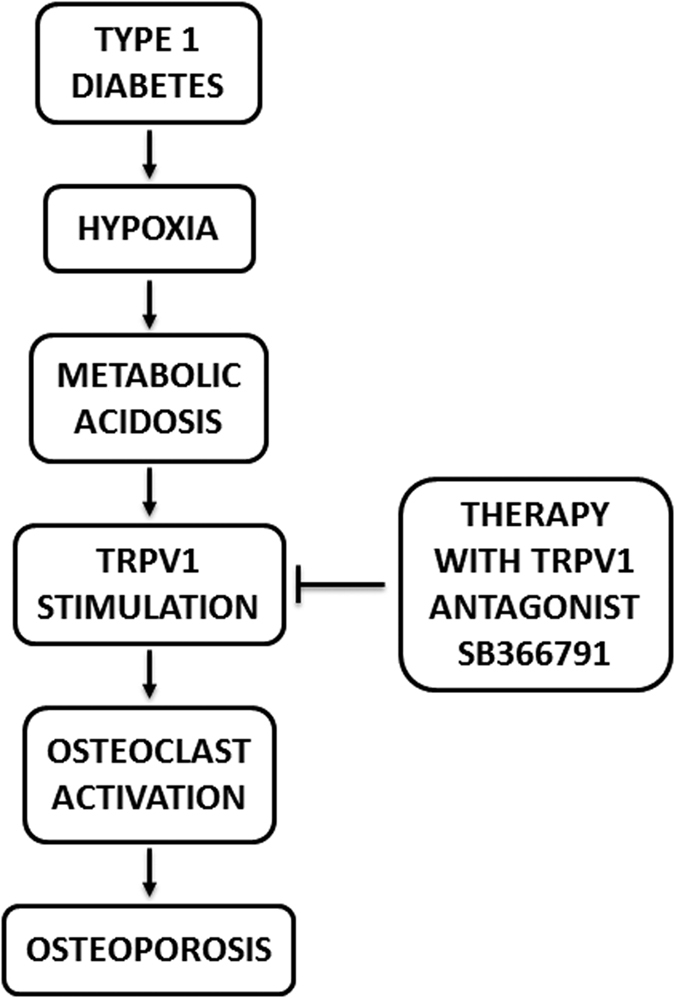
Schematic representation showing the proposed mechanisms for the development of osteoporosis in the setting of DM1. Type 1 diabetes induces hypoxia in the bone marrow, which determines local metabolic acidosis. The decrease in pH stimulates the expression and activation of the polymodal cation channel TRPV1 which, in turn, promotes the activation of osteoclasts, leading to osteoporosis. Therapies aimed at preventing TRPV1 stimulation (ie. by using the TRPV1 antagonist SB366791) would prevent the increased osteoclast activation observed in the setting of DM1, thus protecting the diabetic bone from osteoporosis.

Using a murine model of DM1, we demonstrate the activation of osteoclasts in trabecular bone since early stages of the disease. Ostoclastogenesis is mainly attributable to local acidosis, which triggers the non-canonical TRPV1 pathway. Importantly, inhibition of this pathway by using SB-366791, a potent and selective TRPV1 antagonist, is sufficient to prevent osteoclast activation both *in vitro* and *in vivo* (Fig. 8).

The main players involved in osteoclast differentiation and activation under physiological condition are RANKL, produced by stromal cells, and its decoy receptor OPG^15^. Binding of RANKL to its receptor RANK on osteoclast precursors promotes cell differentiation through the activation of Akt, NFκB and MAPK pathways^15^. Intriguingly we found that this canonical pathway is not involved in DM1-induced osteoclast activation, implying a non-canonical mechanism. Monocytes and osteoclast precursors express β2 integrins such as CD11b/CD18 (also termed αMβ2, CR3 or Mac1). CD11b expression is dynamically regulated during murine osteoclastogenesis and has been reported to act as a negative regulator of the earliest stages of osteoclast differentiation, by interfering with RANKL signalling^18^. Evaluation of CD11b/CD18 expression ruled out this mechanism in DM1-induced osteoclast activation.

Previous observations demonstrate that DM severely alters the BM microenvironment inducing a form of microangiopathy, especially at the level of the endosteal region^19^. Microvascular abnormalities were confirmed in a recent study conducted in DM patients with or without peripheral macrovascular disease^20^. Microvascular rarefaction alters the path-length for oxygen and nutrient diffusion across the marrow. Hypoxia is an acknowledged inducer of genes involved in osteoclastogenesis^21^ and is thought to act through HIF-1, but this mechanism remains debated^21^. In particular, a recent study reported that the expression of a constitutively active form of HIF-1 actually attenuates osteoclast differentiation^22^. In line, when examining the effect of the hypoxia mimetic DMOG, which enhances HIF-1 levels by inhibiting prolyl-4-hydroxylase domain enzymes, we observed an inhibition of osteoclast differentiation. Alternatively, hypoxia may induce the secretion of inflammatory cytokines like TNF-α, IL1-α and IL1-β^23^, which together with RANKL cooperatively orchestrate osteoclastogenesis^24^. However, the protein expression of TNF-α, IL1-α and IL1-β was not affected by high glucose and hypoxic conditions. Intriguingly, we observed that hypoxia determines a reduction of the pH of cell media, which led us to consider acidosis as the mechanism involved in osteoclast activation^21^,^25^. Accordingly, pH stabilization of *in vitro* hypoxic culture conditions prevented osteoclast differentiation, thus pinpointing acidosis as a possible key factor. This was confirmed by comparing the pH of PB and BM supernatants from diabetic and control mice. Nevertheless, a rescue experiment by using insulin implants to achieve metabolic control failed to correct local acidosis and osteoclastogenesis in diabetic mice. This data could explain the persistence of increased risk of bone fractures observed in diabetic patients with achieved metabolic control^1^,^2^ and correlates with a clinical study showing increased bone turnover during DM1^26^.

Acidosis is known to induce the formation and activation of osteoclasts through the stimulation of two different TRP cation channels, TRPV1 and TRPV4^16^,^17^. TRPV1 is a polymodal receptor and a nonselective cation channel that may be activated by heat, low pH and some pungent chemicals such as capsaicin and alliyl isothiocyanate. In addition to its location in the peripheral^27^ and central^28^ nervous system, TPVR1 is expressed in all “gate” barrier tissues and peripheral non-neuronal tissues of rodents^29^. In addition, TRPV1 has been identified in monocytes^16^,^30^. However, the role of TRPV1 in the BM and its implication in diabetic osteoporosis has never been described before. In this study, we demonstrate, for the first time, an increase of TRPV1 in the diabetic BM. Using gain-and loss-of-function approaches *in vitro*, we confirm that acidosis triggers osteoclast activation through the modulation of TRPV1. The involvement of TRPV1 in DM1-induced osteoclast activation was further established *in vivo* using prolonged administration of a TRPV1 receptor antagonist. This new finding opens new therapeutic avenues for the treatment of a common, life-threating pathology frequently associated with DM1. Correction of local acidosis in the BM might prove to be extremely challenging due to difficulties in reaching the BM compartment, whereas the systemic administration of TRPV1 antagonist to reduce osteoclast activation is clinically possible. TRPV1 antagonists have in fact already advanced to clinical trials as analgesics^31^. Furthermore, TRPV1 antagonists have therapeutic indications in the treatment of DM2, because of their dual effects as insulin sensitizers and secretagogues^32^ and as anti-obesity drugs^33^ studies are however necessary to determine the implication of TRPV1 in osteoclastogenesis induced by DM2.

Current therapies for the treatment of osteoporosis and other conditions that exhibit increased osteoclast activation are mainly based on the use of bisphosphonates, as bisphosphonates trigger osteoclasts apoptosis, thereby delaying bone loss^34^. Thus, given their inhibitory effect on osteoclast activation, TRPV1 antagonists may represent an alternative strategy for the treatment of osteoporosis.

In conclusion, our results highlight new mechanistic understanding of precocious osteoporosis in DM and open potential therapeutic avenues, such as TRPV1 antagonists, for treatment of bone pathology in patients with DM.

## Material and Methods

### Animal studies

The experiments involving mice received the approval of the UK Home Office and the University of Bristol and were performed in accordance with the Guide for Care and Use of Laboratory Animals prepared by the Institute of Laboratory Animal Resource. DM1 was induced in male CD1 mice (5–6 week-old, Harlan, UK) by injection of STZ (40 mg/kg body weight IP, daily for 5 days; Sigma-Aldrich, Saint Luis, USA)^19^,^35^. DM1 was confirmed by regular measurements of urine glucose levels (Diastix, Bayer, Leverkusen, Germany). Animals with glucose levels >2000 mg/dL were considered diabetic. Sex- and age-matched CD1 non-diabetic mice injected with the vehicle of STZ (citrate buffer, pH 4.5) were used as controls. Analyses were performed at 5 and 11 weeks after DM induction.

Two studies were conducted to prevent osteoclast activation in STZ-induced diabetic mice. In a first set of experiments, metabolic control of DM was considered. To this aim, mice were implanted subcutaneously with a sustained-release insulin implant (LinBit, LinShin, Toronto, Canada). The implants provide the mice with insulin for up to 5 weeks. Implanted animals showed negligible urinary glucose levels. In a second set, TRPV1 receptors were blocked by IP injections of the TRPV1 receptor antagonist SB366791 (1 mg/kg, Tocris Bioscience, Bristol, UK)^36^ for 14 consecutive days. Control mice received vehicle instead of SB366791.

### Histochemical analyses

Mice were sacrificed by neck dislocation under terminal anesthesia (Avertin, Sigma-Aldrich) and femurs devoid of muscle and connective tissue were collected and fixed in 4% paraformaldehyde for 24 hrs at 4 °C. Bones were decalcified in 10% formic acid over-night. Paraffin embedded samples were cut (5 μm thickness) for subsequent histochemical analyses. Active osteoclasts were detected by TRAP staining (Acid phosphatase, leukocyte kit, Sigma-Aldrich). Sections were counterstained with Hematoxylin. Positive osteoclasts were identified as multinucleated brown/red cells lining the bone surface in both metaphyseal and epiphyseal regions. The number of active osteoclasts was normalized by length of the bone surface (in mm) using an image-analysis software (Image ProPlus, Media Cybernetics).

Histological sections stained with Hematoxylin and Eosin were used to quantify the area occupied by the bone per tissue area in both metaphyseal and epiphyseal regions.

### Isolation of BM cells

Tibias were flushed with DMEM. BM cells were separated by a gentle dispersion. The remaining bones were crushed into a sterile mortar and digested following manufacture’s instructions (Bone Marrow Harvesting & Hematopoietic Stem Cell Isolation Kit, Millipore, Billerica, USA) in order to isolate cells from the endosteal region.

### BM Mononuclear Cells Culture

BM cells were collected as described above and cultured for 24 hs in α-MEM/10% FBS to allow attachment of stromal cells to the plate. Supernatant containing non-adherent BM-MNCs was collected, and after red blood cell lysis, cells were re-plated in differentiation media (α-MEM 10%FBS, RANKL 70 ng/ml and M-CSF 10 ng/ml) for 6 days. Media was changed every 3 days^37^. To mimic the BM microenvironment during DM, BM-MNCs cells were cultured in α-MEM/10%FBS with high glucose (25 mM glucose) and/or under hypoxic condition (PPO_2_ 2%), dimethyloxaloylglycine (DMOG, Sigma-Aldrich) or with TRPV1 agonists (capsaicin or olvanil; 10 μM, Tocris Bioscience) or antagonists (capsazepine or SB 366791, 10 μM, Tocris Bioscience) and under acidosis (pH 7.2). To stabilise pH in hypoxic conditions, 20 mM of HEPES (Sigma-Aldrich), was added to culturing media. Manipulation and adjustments of pH were performed by addition of small amounts of NaOH or HCl to culture medium.

### Measurement of BM pH

Femurs and tibias were air flushed and BM pH immediately measured with a micro pH electrode (Eutech Instruments, Landsmeer, The Netherlands) connected to a pH meter.

### Cytochemistry and immunocytochemistry

BM-MNCs were differentiated as previously described and fixed in 4% PFA, 10 min at RT. Osteoclast differentiation was evaluated by TRAP staining (1 hr at 37 °C). Nuclei were counterstained with Hematoxylin. TRAP staining was evaluated under inverted microscope (Axiovert 200, Zeiss, Oberkochen, Germany). Positive osteoclasts were identified as giant brown/red cells with 3 or more nuclei. TRPV1 was additionally evaluated in differentiated BM-MNCs. Briefly, BM-MNCs were fixed (4% PFA), nonspecific staining blocked with 1% Goat Serum/0.1% Triton-X in PBS and incubated overnight at 4 °C with a rabbit polyclonal antibody against TRPV1 (Abcam). Cells were then incubated with Goat Anti-Rabbit Alexa-Fluo 488 secondary antibody (Invitrogen) 1 hr at RT. Nuclei were counterstained with DAPI (Sigma-Aldrich). Images were captured at 400X magnification. Fluorescence intensity was quantified using image J software as previously described^38^.

### ELISA assays

Bones were collected as described above. BM cells were flushed with 200 μl of PBS. BM supernatant was obtained by centrifugation (300 *g* for 10′ at 4 °C. PB (500 ul) was collected from beating hearts of mice. RANKL and OPG protein levels in mouse PB plasma and BM supernatant was performed using “Quantikine Mouse TRANCE/RANK-Ligand/TNFSF11 Immunoassay and Mouse OPG/TNFRSF11B Immunoassay” (R&D System, Minneapolis, USA) following manufacturer’s instructions.

Degradation products from C-terminal telopeptides of type I collagen (CTX-I) and osteocalcin levels were measured in mouse PB plasma using “RatLaps (CTX-1) EIA” (Immunodiagnostic Systems, UK) and “Gla-osteocalcin high sensitive EIA” (Clontech, France) respectively.

### Flow Cytometry

Freshly harvested BM cells were stained for CD11b (BD Bioscience, San Jose, USA). Flow cytometry was performed on FACSCanto II and FACSLSRII equipped with FACSDiva software (BD Biosciences).

### Western blot

Proteins were extracted from BM cells (isolated as described above) by using ice-cold lysis buffer (50 mM Hepes pH 7.5; 150 mM NaCl; 1 mM EDTA; 25 mM EGTA; 25 mM NaF; 5 mM NaPPi; 1% Triton-X; 1% NP-40; 0.25% sodium deoxycholate, protease and phosphatase inhibitors). Proteins were transferred to PVDF membranes (Bio-Rad Laboratories) and probed with primary antibodies against TRPV1 (Abcam, Cambridge,UK) and β-Tubulin (Cell Signaling, Danvers, USA) followed by HRP- conjugated secondary anti-rabbit goat IgG (Sigma-Aldrich). Detection was developed by chemiluminescence reaction (ECL, GE Healthcare, Little Chalfont, UK).

### Statistical Analysis

Results are presented as means ± SEM. Differences among multiple groups were compared by analysis of variance (1- or 2-way ANOVA and Bonferroni post-hoc test), and differences between 2 groups were compared by paired or unpaired Student t test. A P value <0.05 was interpreted to denote statistical significance. Stated n values represent biological replicates.

## Additional Information

**How to cite this article**: Reni, C. *et al.* Diabetes Stimulates Osteoclastogenesis by Acidosis-Induced Activation of Transient Receptor Potential Cation Channels. *Sci. Rep.* **6**, 30639; doi: 10.1038/srep30639 (2016).

## Supplementary Material

Supplementary Information

## References

[b1] Räkel A., Sheehy O., Rahme E. & LeLorier J. Osteoporosis among patients with type 1 and type 2 diabetes. Diabetes Metab. 34, 193–205 (2008). 18308607 10.1016/j.diabet.2007.10.008

[b2] Hofbauer L. C., Brueck C. C., Singh S. K. & Dobnig H. Osteoporosis in patients with diabetes mellitus. J. Bone Miner. Res. 22, 1317–1328 (2007). 17501667 10.1359/jbmr.070510

[b3] Moseley K. F. Type 2 diabetes and bone fractures. Curr Opin Endocrinol Diabetes Obes 19, 128–135 (2012). 22262002 10.1097/MED.0b013e328350a6e1 PMC4753802

[b4] Leslie W. D., Rubin M. R., Schwartz A. V. & Kanis J. A. Type 2 diabetes and bone. J. Bone Miner. Res. 27, 2231–2237 (2012). 23023946 10.1002/jbmr.1759

[b5] Burge R. *et al.* Incidence and economic burden of osteoporosis-related fractures in the United States, 2005–2025. J. Bone Miner. Res. 22, 465–475 (2007). 17144789 10.1359/jbmr.061113

[b6] Alikhani M. *et al.* Advanced glycation end products stimulate osteoblast apoptosis via the MAP kinase and cytosolic apoptotic pathways. Bone 40, 345–353 (2007). 17064973 10.1016/j.bone.2006.09.011 PMC1913208

[b7] Weinberg E., Maymon T., Moses O. & Weinreb M. Streptozotocin-induced diabetes in rats diminishes the size of the osteoprogenitor pool in bone marrow. Diabetes Res. Clin. Pract. 103, 35–41 (2014). 24314392 10.1016/j.diabres.2013.11.015

[b8] Orlandi A. *et al.* Long-term diabetes impairs repopulation of hematopoietic progenitor cells and dysregulates the cytokine expression in the bone marrow microenvironment in mice. Basic Res Cardiol 105, 703–712 (2010). 20652278 10.1007/s00395-010-0109-0

[b9] Hie M., Shimono M., Fujii K. & Tsukamoto I. Increased cathepsin K and tartrate-resistant acid phosphatase expression in bone of streptozotocin-induced diabetic rats. Bone 41, 1045–1050 (2007). 17916452 10.1016/j.bone.2007.08.030

[b10] Kayal R. A. *et al.* Diminished bone formation during diabetic fracture healing is related to the premature resorption of cartilage associated with increased osteoclast activity. J. Bone Miner. Res. 22, 560–568 (2007). 17243865 10.1359/jbmr.070115 PMC3109431

[b11] Zayzafoon M., Stell C. & Irwin R. Extracellular glucose influences osteoblast differentiation and c–jun expression. Journal of cellular … (2000). 10.1002/1097-4644(20001101)79:2<301::aid-jcb130>3.0.co;2-0 10967557

[b12] Wittrant Y. *et al.* High d(+)glucose concentration inhibits RANKL-induced osteoclastogenesis. Bone 42, 1122–1130 (2008). 18378205 10.1016/j.bone.2008.02.006 PMC2696157

[b13] Xu F. *et al.* Inhibitory effects of high glucose/insulin environment on osteoclast formation and resorption *in vitro*. J. Huazhong Univ. Sci. Technol. Med. Sci. 33, 244–249 (2013). 23592138 10.1007/s11596-013-1105-z

[b14] Vestergaard P. Discrepancies in bone mineral density and fracture risk in patients with type 1 and type 2 diabetes–a meta-analysis. Osteoporos Int 18, 427–444 (2007). 17068657 10.1007/s00198-006-0253-4

[b15] Boyle W. J., Simonet W. S. & Lacey D. L. Osteoclast differentiation and activation. Nature 423, 337–342 (2003). 12748652 10.1038/nature01658

[b16] Idris A. I., Landao-Bassonga E. & Ralston S. H. The TRPV1 ion channel antagonist capsazepine inhibits osteoclast and osteoblast differentiation *in vitro* and ovariectomy induced bone loss *in vivo*. Bone 46, 1089–1099 (2010). 20096813 10.1016/j.bone.2010.01.368

[b17] Lieben L. & Carmeliet G. The Involvement of TRP Channels in Bone Homeostasis. Front Endocrinol (Lausanne) 3, 99 (2012). 22934090 10.3389/fendo.2012.00099 PMC3422722

[b18] Park-Min K.-H. *et al.* Negative regulation of osteoclast precursor differentiation by CD11b and β2 integrin-B-cell lymphoma 6 signaling. J. Bone Miner. Res. 28, 135–149 (2013). 22893614 10.1002/jbmr.1739 PMC3522783

[b19] Oikawa A. *et al.* Diabetes mellitus induces bone marrow microangiopathy. Arterioscler Thromb Vasc Biol 30, 498–508 (2010). 20042708 10.1161/ATVBAHA.109.200154 PMC3548136

[b20] Spinetti G. *et al.* Global remodeling of the vascular stem cell niche in bone marrow of diabetic patients: implication of the microRNA-155/FOXO3a signaling pathway. Circ Res 112, 510–522 (2013). 23250986 10.1161/CIRCRESAHA.112.300598 PMC3616365

[b21] Arnett T. R. Acidosis, hypoxia and bone. Arch Biochem Biophys 503, 103–109 (2010). 20655868 10.1016/j.abb.2010.07.021

[b22] Leger A. J. *et al.* Inhibition of osteoclastogenesis by prolyl hydroxylase inhibitor dimethyloxallyl glycine. J Bone Miner Metab 28, 510–519 (2010). 20300790 10.1007/s00774-010-0171-6

[b23] Li Z., Wei H., Deng L., Cong X. & Chen X. Expression and secretion of interleukin-1β, tumour necrosis factor-α and interleukin-10 by hypoxia- and serum-deprivation-stimulated mesenchymal stem cells. FEBS Journal 277, 3688–3698 (2010). 20681988 10.1111/j.1742-4658.2010.07770.x

[b24] Osta B., Benedetti G. & Miossec P. Classical and Paradoxical Effects of TNF-α on Bone Homeostasis. Front Immunol 5, 48 (2014). 24592264 10.3389/fimmu.2014.00048 PMC3923157

[b25] Kato. Promotion of osteoclast differentiation and activation in spite of impeded osteoblast-lineage differentiation under acidosis: Effects of acidosis on bone metabolism. Biosci Trends doi: 10.5582/bst.2013.v7.1.33 (2013). 23524891

[b26] Gallacher S. J. *et al.* An evaluation of bone density and turnover in premenopausal women with type 1 diabetes mellitus. Diabet Med 10, 129–133 (1993). 8096168 10.1111/j.1464-5491.1993.tb00029.x

[b27] Caterina M. J. & Julius D. The vanilloid receptor: a molecular gateway to the pain pathway. Annu. Rev. Neurosci. 24, 487–517 (2001). 11283319 10.1146/annurev.neuro.24.1.487

[b28] Jancsó-Gábor A., Szolcsányi J. & Jancsó N. Stimulation and desensitization of the hypothalamic heat-sensitive structures by capsaicin in rats. J. Physiol. (Lond.) 208, 449–459 (1970). 5500735 10.1113/jphysiol.1970.sp009130 PMC1348759

[b29] Gunthorpe M. J. & Szallasi A. Peripheral TRPV1 receptors as targets for drug development: New molecules and mechanisms. Curr Pharm Des 14, 32–41 (2008). 18220816 10.2174/138161208783330754

[b30] Khan K. *et al.* [6]-Gingerol induces bone loss in ovary intact adult mice and augments osteoclast function via the transient receptor potential vanilloid 1 channel. Mol Nutr Food Res 56, 1860–1873 (2012). 23034900 10.1002/mnfr.201200200

[b31] Lambert D. G. Capsaicin receptor antagonists: a promising new addition to the pain clinic. Br J Anaesth 102, 153–155 (2009). 19151045 10.1093/bja/aen354

[b32] Tanaka H. *et al.* Enhanced insulin secretion and sensitization in diabetic mice on chronic treatment with a transient receptor potential vanilloid 1 antagonist. Life Sciences 88, 559–563 (2011). 21277869 10.1016/j.lfs.2011.01.016

[b33] Comerma-Steffensen S., Grann M., Andersen C. U., Rungby J. & Simonsen U. Cardiovascular effects of current and future anti-obesity drugs. Curr Vasc Pharmacol 12, 493–504 (2014). 24846238 10.2174/1570161112666140423223529

[b34] Weinstein R. S., Roberson P. K. & Manolagas S. C. Giant osteoclast formation and long-term oral bisphosphonate therapy. N Engl J Med 360, 53–62 (2009). 19118304 10.1056/NEJMoa0802633 PMC2866022

[b35] Mangialardi G. *et al.* Diabetes causes bone marrow endothelial barrier dysfunction by activation of the RhoA-Rho-associated kinase signaling pathway. Arterioscler Thromb Vasc Biol 33, 555–564 (2013). 23307872 10.1161/ATVBAHA.112.300424 PMC3616369

[b36] Varga A. *et al.* Effects of the novel TRPV1 receptor antagonist SB366791 *in vitro* and *in vivo* in the rat. Neurosci. Lett. 385, 137–142 (2005). 15950380 10.1016/j.neulet.2005.05.015

[b37] Orriss I. R. & Arnett T. R. Rodent osteoclast cultures. Methods Mol. Biol. 816, 103–117 (2012). 22130925 10.1007/978-1-61779-415-5_8

[b38] Gavet O. & Pines J. Activation of cyclin B1-Cdk1 synchronizes events in the nucleus and the cytoplasm at mitosis. J. Cell Biol. 189, 247–259 (2010). 20404109 10.1083/jcb.200909144 PMC2856909

