# Supplementary material for: Diabetes Stimulates Osteoclastogenesis by Acidosis-Induced Activation of Transient Receptor Potential Cation Channels
Source: Sci Rep. 2016 Jul 29;6:30639. doi: 10.1038/srep30639 (PMC4965751; doi:10.1038/srep30639)
Supplement: Supplementary Information [file srep30639-s1.pdf]

Online Supplemental Material

Diabetes Stimulates Osteoclastogenesis by Acidosis-Induced Activation of Transient Receptor Potential Cation Channels

Carlotta Reni, Giuseppe Mangialardi, Marco Meloni, Paolo Madeddu

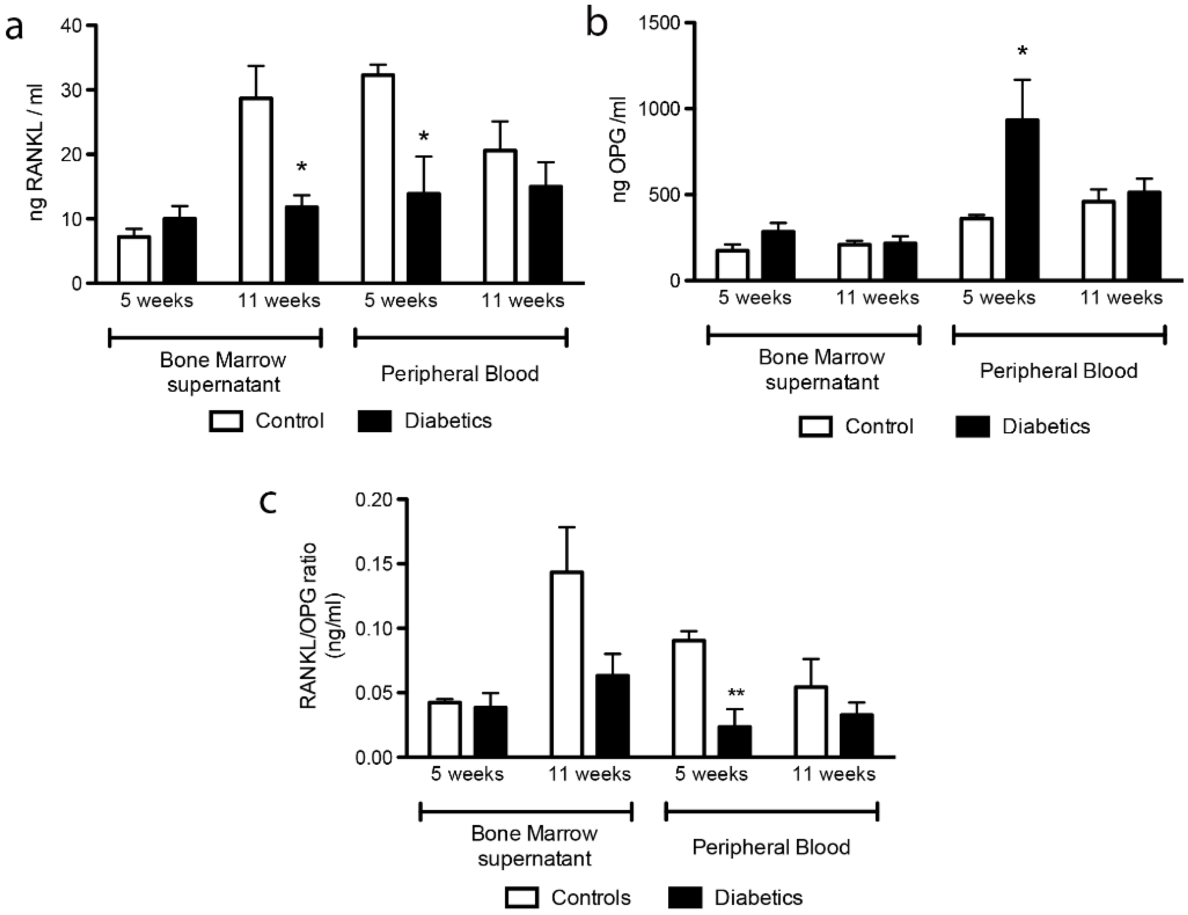

Figure S1: DM-induced osteoclast activation is RANKL/OPG independent. ELISA assay show changes in the expression of RANKL (a) and OPG (b) in mouse BM supernatants and PB samples collected at 5 and 11 weeks post-DM induction as compared to control mice. Bar graphs show the RANKL/OPG ratio in the BM and PB (c) at both time points. Data are expressed as mean±SEM. *p<0.05 vs non-diabetic controls at 5 weeks; **p<0.01 vs non-diabetic controls. n=4 samples/group.

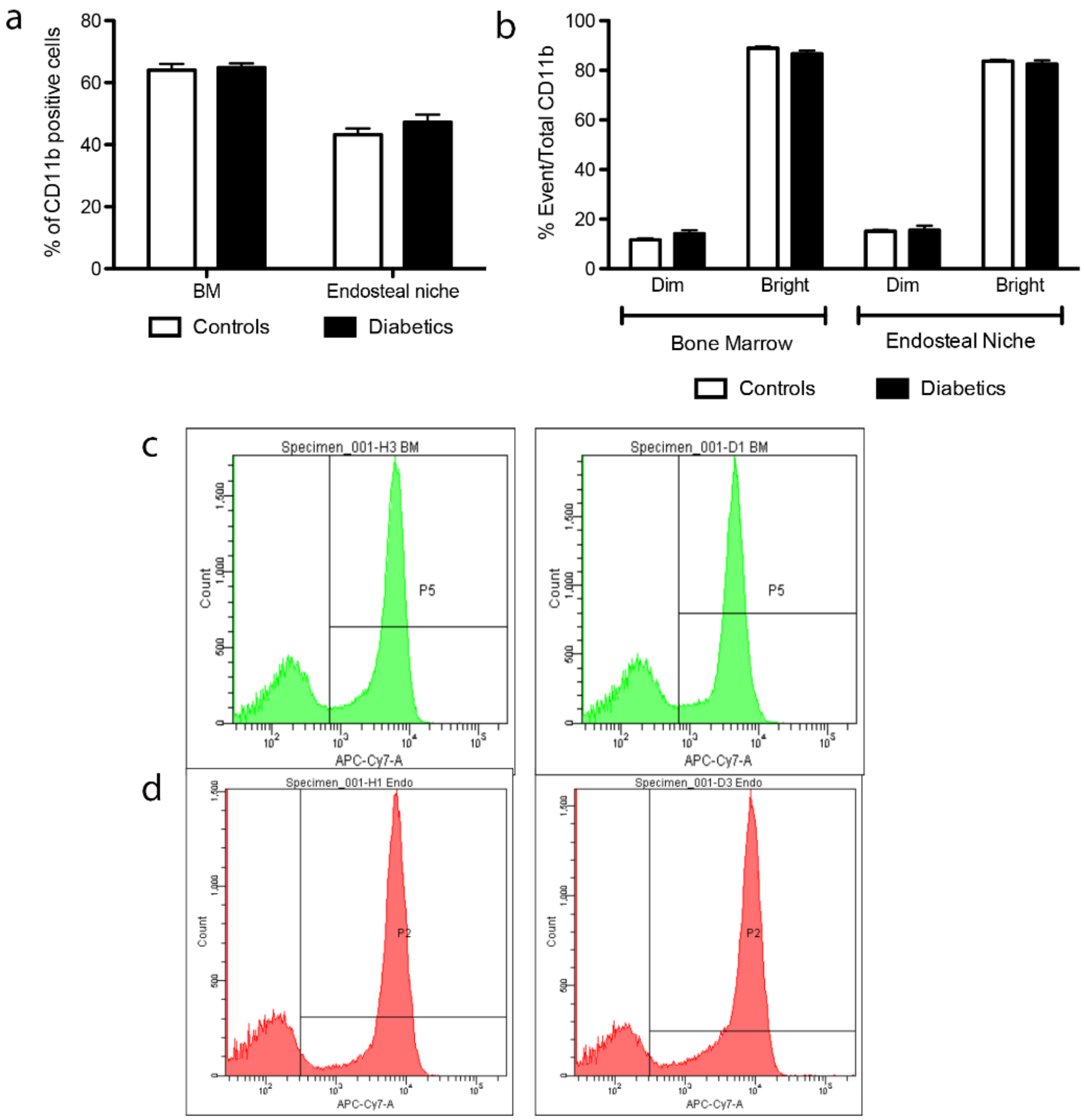

Figure S2: DM does not modify the number of osteoclast precursors in the BM. Bar graph a shows the percentage of BM CD11b positive cells from central and endosteal regions of controls and diabetic animals. Bar graph b shows the percentage of dim and bright populations among the CD11b positive cells from central and endosteal regions of control and diabetic animals. Representative histograms show the intensity of CD11b signal in cells from central (c) and endosteal regions (d) in control (left panels) and diabetic (right panels) mice. Data are expressed as mean±SEM. n=5 samples/group.
